# Default Mode Network Modulation by Psychedelics: A Systematic Review

**DOI:** 10.1093/ijnp/pyac074

**Published:** 2022-10-22

**Authors:** James J Gattuso, Daniel Perkins, Simon Ruffell, Andrew J Lawrence, Daniel Hoyer, Laura H Jacobson, Christopher Timmermann, David Castle, Susan L Rossell, Luke A Downey, Broc A Pagni, Nicole L Galvão-Coelho, David Nutt, Jerome Sarris

**Affiliations:** MDHS, University of Melbourne, Parkville, Victoria, Australia; Florey Department of Neuroscience and Mental Health, University of Melbourne, Parkville, Victoria, Australia; Psychae Institute, Melbourne, Victoria, Australia; MDHS, University of Melbourne, Parkville, Victoria, Australia; School of Social and Political Science, University of Melbourne, Australia; Centre for Mental Health, Swinburne University, Hawthorn, Victoria, Australia; The Institute of Psychiatry, Psychology and Neuroscience, King’s College London, UK; Florey Department of Neuroscience and Mental Health, University of Melbourne, Parkville, Victoria, Australia; MDHS, University of Melbourne, Parkville, Victoria, Australia; Florey Department of Neuroscience and Mental Health, University of Melbourne, Parkville, Victoria, Australia; The Scripps Research Institute, Department of Molecular Medicine, La Jolla, California, USA; MDHS, University of Melbourne, Parkville, Victoria, Australia; Florey Department of Neuroscience and Mental Health, University of Melbourne, Parkville, Victoria, Australia; Centre for Psychedelic Research, Division of Psychiatry, Imperial College London, UK; Department of Psychiatry, University of Toronto, Canada; Centre for Mental Health, Swinburne University, Hawthorn, Victoria, Australia; Centre for Human Psychopharmacology, Swinburne University, Hawthorn, Victoria, Australia; College of Health Solutions, Arizona State University, Tempe, Arizona, USA; Department of Physiology and Behavior, Universidade Federal do Rio Grande do Norte, Brazil; NICM Health Research Institute, Western Sydney University, Westmead, New South Wales, Australia; Centre for Psychedelic Research, Division of Psychiatry, Imperial College London, UK; Psychae Institute, Melbourne, Victoria, Australia; Florey Department of Neuroscience and Mental Health, University of Melbourne, Parkville, Victoria, Australia; NICM Health Research Institute, Western Sydney University, Westmead, New South Wales, Australia

**Keywords:** Psychedelics, psilocybin, LSD, ayahuasca, connectivity, DMN

## Abstract

Psychedelics are a unique class of drug that commonly produce vivid hallucinations as well as profound psychological and mystical experiences. A grouping of interconnected brain regions characterized by increased temporal coherence at rest have been termed the Default Mode Network (DMN). The DMN has been the focus of numerous studies assessing its role in self-referencing, mind wandering, and autobiographical memories. Altered connectivity in the DMN has been associated with a range of neuropsychiatric conditions such as depression, anxiety, post-traumatic stress disorder, attention deficit hyperactive disorder, schizophrenia, and obsessive-compulsive disorder. To date, several studies have investigated how psychedelics modulate this network, but no comprehensive review, to our knowledge, has critically evaluated how major classical psychedelic agents—lysergic acid diethylamide, psilocybin, and ayahuasca—modulate the DMN. Here we present a systematic review of the knowledge base. Across psychedelics there is consistent acute disruption in resting state connectivity within the DMN and increased functional connectivity between canonical resting-state networks. Various models have been proposed to explain the cognitive mechanisms of psychedelics, and in one model DMN modulation is a central axiom. Although the DMN is consistently implicated in psychedelic studies, it is unclear how central the DMN is to the therapeutic potential of classical psychedelic agents. This article aims to provide the field with a comprehensive overview that can propel future research in such a way as to elucidate the neurocognitive mechanisms of psychedelics.

## INTRODUCTION

Psychedelics are a class of hallucinogenic agents that have cultural, spiritual, and scientific implications (McKenna, 1998; Nichols, 2016). The etymology of the term psychedelic is from the Greek words ψυχή (psyche, “soul, mind”) and δηλοῦν (deloun, “to manifest”). Therefore, psychedelics are “mind/soul manifesting” and are suggested to illuminate hidden terrains of the human psyche (Weil and Winifred, 2004). “Classical psychedelics” is a broad term describing a variety of substances whose primary mechanism of action resides in serotonin (5-HT_2A_) receptors in the brain and, in turn, produce profound alterations of consciousness, including modulations in the sense of self, sensory perception, and emotions (Table 1) (Morris, 2008; Nichols, 2016; Speth et al., 2016).

**Table 1.** Structure and Function of the Classical Psychedelics, Their Ability to Modulate the DMN, and Associated Therapeutic Outcomes

Psychedelic	Neuropharmacology	Subjective effects	DMN modulation	Therapeutic effects	Relevant studies
Psilocybin 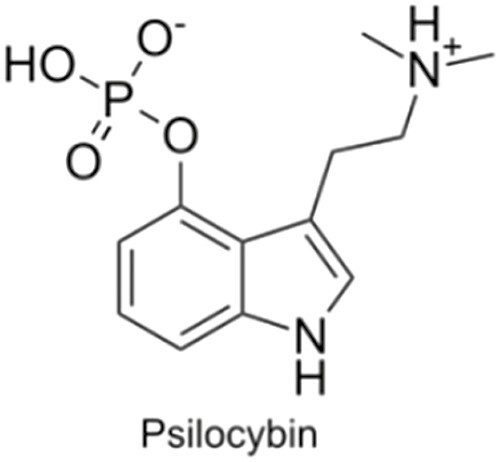 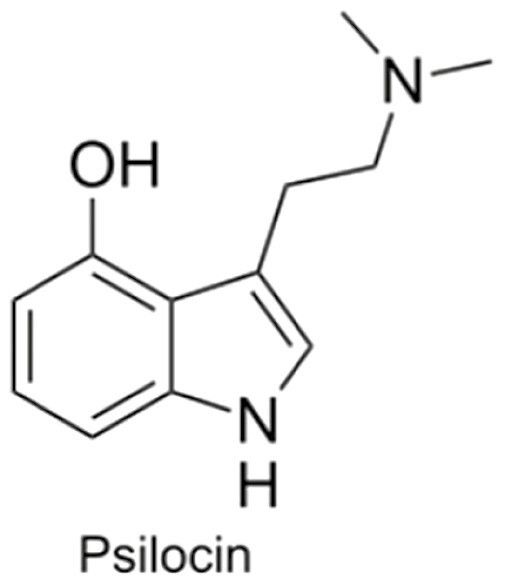	Psilocybin is tryptamine derivative, and its chemical structure is comprised of an indole ring linked to an ethylamine substituent. Upon ingestion of psilocybin, it is dephosphorylated to psilocin, which is an agonist of several serotonin receptors with its highest affinity for 5-HT_1_ and 5-HT_2_ receptors.	Psilocybin significantly impacts time distortion, where users feel that “time is standing still.” The experience usually lasts 4–6 h. Users having a pleasant experience generally have a feeling of euphoria, oneness, and connectedness. However, an unpleasant experience can also occur, which is usually characterized by intense fear, anxiety, and distress.	Psilocybin robustly decreases FC within the DMN and increases global brain connectivity. Specifically, decoupling between key DMN nodes such as the mPFC and the PCC has been documented. Furthermore, psilocybin has been reported to decrease DMN integration and reduce local modularity and segregation up to 3 wk after administration in depressed patients.	Psilocybin has been shown to reduce end-of-life anxiety in patients who were terminally ill, improve smoking and alcohol cessation, reduce cluster headaches, and decrease symptoms of depression and PTSD.	(Sewell et al., 2006; Wittmann et al., 2007; Carhart-Harris et al., 2012; Nichols, 2016; Preller and Vollenweider, 2016; Nour and Carhart-Harris, 2017; Thomas et al., 2017; Smigielski et al., 2019; Daws et al., 2022; Mason et al., 2020a; Mertens et al., 2020; Grandjean et al., 2021)
Ayahuasca 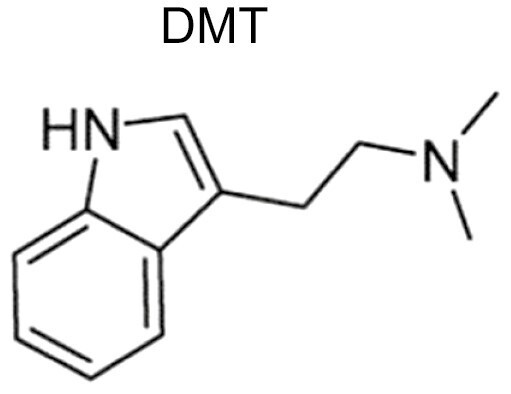 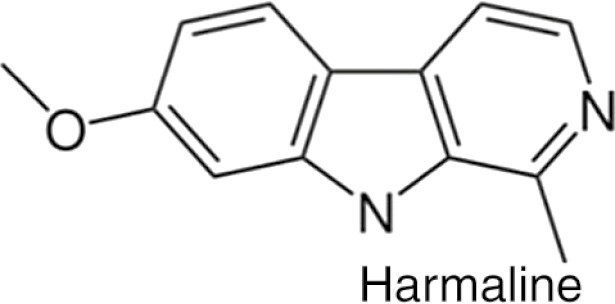	Ayahuasca is a psychoactive decoction and is usually made from the *Banisteriopsis caapi* vine, the *Psychotria viridis* shrub. The *B. caapi* vine, importantly contains harmala alkaloids, which render the hallucinogenic component DMT orally active.	Individuals who have consumed ayahuasca often report it as being a profound spiritual and mystical experience. Often people feel more connected to nature and the earth and start to understand their place in the world. The experience can last 2-6 h and can involve “purging,” which is characterised by vomiting and diarrhea.	In a similar fashion to other psychedelics, ayahuasca disrupts normal DMN function during the psychedelic experience. It has been shown that long-term ayahuasca use has led to increased thickness of the ACC and decreased thickness of the PCC (a key DMN node). Finally, ayahuasca increases local and decreases global network integration, while increasing Shannon entropy across networks.	Ayahuasca possesses antidepressant and anxiolytic effects. It has been reported to increase acceptance and trait mindfulness and may have some efficacy for treating addictions and trauma.	(McKenna, 1998; Shanon, 2002; Barbosa et al., 2012; Bouso et al., 2015; Palhano-Fontes et al., 2015; Sampedro et al., 2017; Viol et al., 2017; Uthaug et al., 2018; Murphy-Beiner and Soar, 2020; dos Santos and Hallak, 2021; Zeifman et al., 2021)
LSD 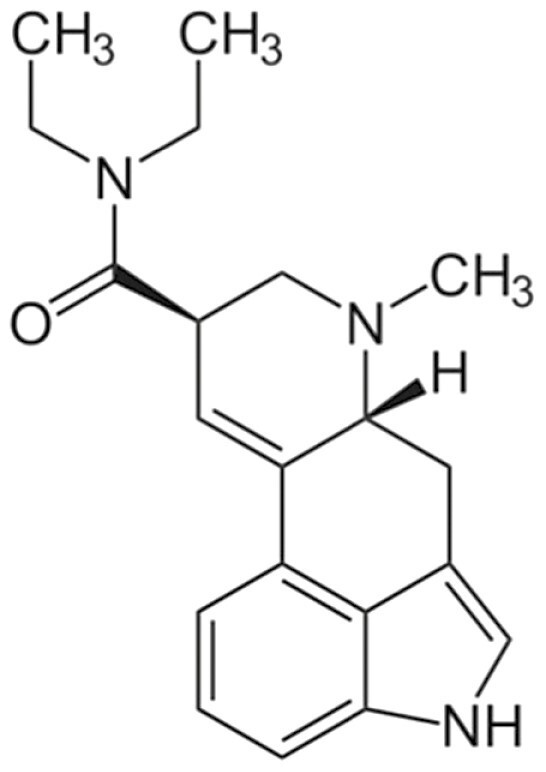	LSD is an ergoline derivative, often synthesised via reactive mechanisms. For instance, diethylamine is reacted via activating reagents with the ergoline alkaloid precursor lysergic acid, forming LSD. LSD binds to both dopamine and serotonin (especially 5-HT_2_ and 5-HT_1_) receptors, although it labels all 5-HT receptors except 5-HT_3_.	Due to the binding of both dopamine and serotonin receptors, the LSD experience generally feels more fast paced and energetic compared with psilocybin. Subjective reports of LSD-induced experiences describe the experience as being highly significant and typically involving intense joy and a sense of spiritual enlightenment. Negative experiences can also occur, however, which include increased paranoia, fear of madness, and anxiety. Experiences last about 6–12 h.	LSD effects the DMN in a remarkably similar way to psilocybin. For instance, LSD decreases within-network connectivity (specifically the medial-posterior DMN) and increases between-network connections.	LSD has been shown to reduce alcoholism, and preliminary evidence shows that it can help reduce symptoms of depression, anxiety, and cluster headaches and may also possess analgesic properties.	(Hofmann, 1980; Sewell et al., 2006; Carhart-Harris et al., 2016; Speth et al., 2016; Tagliazucchi et al., 2016; Atasoy et al., 2017; Müller et al., 2018; Varley et al., 2020; de Gregorio et al., 2021; Nutt and Carhart-Harris, 2021)

Abbreviations: FC, functional connectivity; DMN, default mode network; mPFC, medial prefrontal cortex; PCC, posterior cingulate cortex; PTSD, post-traumatic stress disorder; DMT, dimethyltryptamine; ACC, anterior cingulate cortex; LSD, lysergic acid diethylamide.

**Figure 1.**
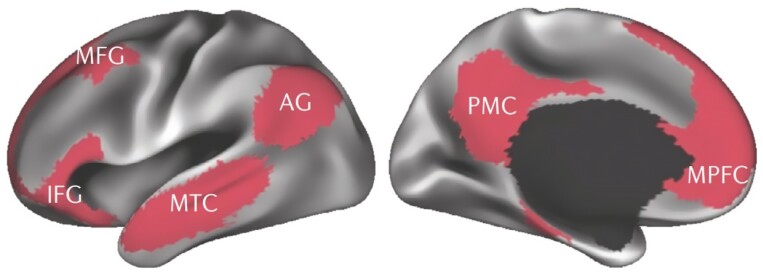
Reprinted by permission from [Springer Nature]: Nature [Nature Reviews Neuroscience]. The default mode network in cognition: a topographical perspective, Smallwood et al. (2021). Brain regions of the DMN based on the coherence of their temporal activity, measured at rest. These regions are the posteromedial cortex (PMC), the medial prefrontal cortex (MPFC), angular gyrus (AG), middle temporal cortex (MTC), middle frontal gyrus (MFG), and inferior frontal gyrus (IFG).

Neuroimaging studies have shown that when an individual is at rest, multiple interconnected brain regions are activated above baseline, and, conversely, there is decreased activity in these regions during task-dependent attention (Raichle, 2015) (Figure 1). These regions amalgamate to create 4 functional hubs: the medial prefrontal cortex (mPFC), the posterior cingulate cortex (PCC), precuneus, and the angular gyrus, together referred to as the Default Mode Network (DMN) (Andrews-Hanna et al., 2014). The DMN is one of many resting-state networks (RSNs). RSNs are derived from the cognitive origin hypothesis of resting-state connectivity, where resting-state connectivity is defined as the synchronous fluctuation of low-frequency signals between functionally related brain areas (Biswal et al., 1997).

Functional connectivity (FC) is defined as the temporal coactivation patterns of neural activity between anatomically distinct brain regions, it is an important and well-established variable of interest when understanding DMN connectivity (van den Heuvel and Pol, 2010). RSNs, such as the DMN, are imaged prior to the experimental stimulus (i.e., engaging in a memory task), and DMN FC has been found to be anti-correlated and orthogonal to task-dependent brain networks such as the salience network (SLN) (Raichle, 2015; Smallwood et al., 2021). Furthermore, altered FC within the DMN has been correlated with a variety of psychometric components in clinical questionnaires, which may have downstream therapeutic effects. For instance, reduced FC with brain regions that comprise the DMN has been associated with positive states of ego dissolution such as Oceanic Self-Boundlessness (measured by the 5-Dimensional Altered States of Consciousness Rating scale; see Table 2), which may facilitate the cognitive reappraisal that can occur during the psychedelic experience (Smigielski et al., 2019; Yaden and Griffiths, 2020).

**Table 2.** Summary of Studies Included for the Systematic Review

Psychedelic	Author/s	Study Design	Sample Characteristics	Outcome Measures	Neuroimaging Technology	Outcome Measures (Results)	Brain Modulation	Limitations/risk of bias
**Psilocybin** (2 mg in 10-mL saline, applied IV)	(Carhart-Harris et al., 2012)	Open-label, within-subjects, Placebo-controlled.	N=15 healthy participants (2 females) were experienced psychedelic drug users Mean age = 32	Intensity of subjective effects which was measured in a visual analogue scale (VAS) format.	3T fMRI (BOLD, ASL, rsfMRI)	All 10 VAS items were scored significantly higher than placebo, suggesting psilocybin had a clear hallucinogenic effect.	**DMN modulation** Decreased CBF and BOLD response in the ACC, PCC, precuneus, bilateral angular cortex and mPFC. **Additional Modulation** Decreased BOLD and blood flow in the putamen, subthalamic nuclei and visual cortex. **Supporting Model** REBUS	Participants had a history of hallucinogenic drug use, which may confound expectations for the placebo condition and the amount (and type of hallucinogen) used previously may vary considerably between individuals affecting reliability and reproducibility. Additionally, there was also a relatively small sample size.
**Psilocybin** (2 mg in 10-mL saline, applied IV)	(Carhart-Harris et al., 2014)	Open-label, within-subjects, Placebo-controlled.	N=15 healthy participants (2 females) were experienced psychedelic drug users Mean age = 32	NA	3T fMRI (BOLD, ASL, rsfMRI)	NA	**DMN modulation** There was significantly greater mean variance of synchrony (a measure of ‘chaos’ or uncertainty) in the DMN in the psilocybin group compared to controls. Using a single patient administered with psilocybin, probability distributions showed that the predictability of the network synchrony was more difficult compared to a placebo control. **Additional modulation** Similarly, to the DMN, increased variance in synchrony was found in the salience and frontoparietal networks. **Supporting Model** REBUS	As the probability distribution was only assessed in one control and treated individual, these findings must be interpreted as exploratory and would need subsequent replication.
**Psilocybin** (10 mg followed by 25 mg, one-week apart)	(Carhart-Harris et al., 2017)	Open-label, within-subjects clinical trial	N = 16 particpants (4 females) diagnosed with treatment resistant depression. Mean age = 43	The Primary Clinical outcome was the 6-item QIDS-SR16	3T fMRI (BOLD, rsfMRI & ASL)	All but one patient showed some decrease in QIDS-SR16 score at week 5 (with one showing no change) and 47% met criteria for a response.	**DMN modulation** Increased DMN integrity one-day post-treatment via both seed and network methodology. However, patients who scored highest on ‘peak’ or ‘mystical’ experiences had the greatest decreases in RSFC in DMN regions such as the PCC. **Additional modulation** Increased vmPFC RSFC with the inferior-lateral parietal cortex (iLPC) post treatment and decreased PH RSFC. Decreased CBF in the left amygdala, Heschl’s gyrus, left planum temporal, and left superior temporal gyrus. **Supporting Model** REBUS	Absence of a placebo-control condition. Correction for multiple testing was not applied to the specific ROI analyses, which can produce a false-positive (type 1 error) result. Additionally, there was also a relatively small sample size.
**Psilocybin** (0.2-0.3 mg/kg, p.o)	(Madsen et al., 2021)	Single blinded repeated measures design.	N = 15 healthy individuals with no psychedelic use within a year of the study. Mean age = 34	11D-ASC, MEQ and EDI	3T fMRI (rsfMRI)	There was a moderate to strong positive correlation (R = 0.57 – 0.6) between subjective drug effects and ASC, MEQ and EDI scores. There was a strong correlation between plasma psilocin levels and subjective drug effects (R^2^ = 0.73)	**DMN modulation** Increased blood psilocin levels were significantly associated with reduced RSFC within the DMN (specifically mPFC, PCC and precuneus) and there was a significant association between increased blood psilocin concentrations and increased between-network average RSFC. **Additional modulation** Local correlation analysis found that blood psilocin levels was significantly negatively correlated with brain regions involved in the executive control network such as the visual cortex, bilateral temporal regions, and the bilateral anterior PFC. **Supporting Model** REBUS	Participants were prepared for a potential strong psychoactive experience by medical professionals which may have produced an expectancy effect and increased the subjective drug effects. Furthermore, a placebo control was not used and the sample size is relatively low, which was partially remedied through a repeated measures design (which increases power).
**Psilocybin** (0.2-0.3mg/kg, p.o)	(McCulloch et al., 2022)	Open-label, within-subjects longitudinal design (mean = 98 days between psilocybin sessions and follow up questionnaires).	N = 10 healthy participants (6 male)	NEO PI-R, MAAS, 11D-ASC, EDI and PEQ.	3T fMRI (rsfMRI) measurments at baseline, 1 week and 3-month time points. PET ([^11^C]Cimbi-36) was used at baseline and 1 week time points.	Participants reported increases in trait openness, mindfulness and increased positive attributes in the PEQ.	**DMN modulation** Psilocybin did not significantly modulate within DMN FC or the FC between the DMN and other networks and a small effect size was observed (*d ≈*0.20). **Additional modulation** Psilocybin statistically decreased the ECN FC at 1 week but not 3 months post administration. Additionally, a moderate-strong negative correlation was found between ECN FC at 1 week and MAAS scores at 3 months. **Supporting model** None	The primary limitation of this study was a small sample size, although authors did report effect sizes. Furthermore, the trial was unblinded (open label) and without a control group.
**Psilocybin** (315 μg/kg, p.o.)	(Smigielski et al., 2019)	Randomized, double-blind, placebo-controlled	N = 38 (23 males) healthy long-term meditators. Mean age = 52	5D-ASC scale was administered to assess acute psychedelic effects. Furthermore, PEQ was administered 4 months after the intervention.	3T fMRI (BOLD & rsfMRI)	Psilocybin significantly increased specific dimensions of the 5D-ASC such as Oceanic Self-Boundlessness, Visionary Restructuralization and Vigilance Reduction. After 4 months the psilocybin group scored significantly higher on the PEQ than the placebo control group.	**DMN Modulation** Significantly increased positive states of ego dissolution (i.e Oceanic self-boundlessness), was associated with the decoupling of FC between the anterior (mPFC) and posterior DMN (PCC) during open awareness meditation for the psilocybin group compared to the placebo condition. **Supporting Model** REBUS	Participants were experienced meditators meaning they may respond uniquely to psychotropic medication, limiting generalisability. Furthermore, the study was conducted in a mindfulness retreat, which may have further bolstered the positive psychological effects which may differ from a traditional lab-based setting.
**Psilocybin** (25 mg/70kg, p.o.)	(Barrett et al., 2020a)	Open-label, within-subjects longitudinal pilot study.	N = 12 (5 male) healthy volunteers who had limited previous use of psychedelics (median = 1). Mean age = 32	Various questionaries were administered one day before, one week after and one month after the psilocybin intervention. These questioners include: PANAS-X, POMS, The Dispositional Positive Emotions Scale (DPES) and the Depression Anxiety Stress Scale (DASS) Finally, participants’ personality traits were measured via The Big Five inventory (BFI)	3T fMRI (rsfMRI & BOLD)	There were significant subacute changes (1 week post treatment) on DASS stress scores, PANAS negative affect, depression, total mood disturbance and trait anxiety. All of these changes except trait anxiety returned to baseline at the one-month post-psilocybin time point.	**DMN Modulation** RSFC was increased across brain networks (including the DMN) and out of a possible 35,778 connections, 695 were significantly found to be different from baseline. **Additional modulation** Negative affect was decreased 1-week post-psilocybin administration and there was decreased amygdala activity in response to emotional stimuli. Additionally, 1-week after the psilocybin ingestion increased responses in reward-learning, attention and decision-based networks were observed. One-month after the initial psilocybin experience increased responses in the somatosensory and fusiform gyrus were measured. **Supporting Model** None	Key limitations which make the findings of this study liable to biases and confounding factors are lack of a placebo or positive control small sample size.
**Psilocybin** (25mg/70kg, p.o.)	(Barrett et al., 2020b)	Single-blinded placebo-controlled	N= 15 healthy long-term meditators (5 female), who had been administered 25mg/70 kg dose in a previous study two months before this initial trial. Mean age =51	Subsequent to each resting state scan participants subjective effects of the intervention were measured via an verbal 11 point scale (0-‘none’, 10 ‘extreme’). Participants were then required to rate three subjective experiences related to mindfulness: presence, letting go and equanimity (emotional poise). Participants also rated their subjective effects against the MEQ and whether they experience has a positive or negative valance.	3T fMRI (BOLD)	Psilocybin increased subjective experiences compared to baseline across various domains. These included 'now-ness’/presence, letting go, awareness, pure-being and pure awareness, timelessness, joy, sacredness and total MEQ scores.	**DMN modulation** Psilocybin significantly decreased FC between the claustrum and the DMN. DMN integrity was correlated with right claustrum connectivity with the DMN. Significantly less FC was observed between the DMN and the right insula **Additional modulation** There was increased connectivity between the claustrum and the FPN. Subjective effects of psilocybin were associated with the amplitude of low-frequency fluctuations and variance within the claustrum. Despite the correlation between subjective effects and neural effects yielding moderate correlation coefficients these associations did not survive multiple comparisons. **Supporting model** CCC	A single dose of psilocybin was assessed and multiple active doses would allow the appropriate dose-dependent analysis to occur. Furthermore, the sample was highly specific as it was comprised of individuals who had a long-term meditation practice and had already received psilocybin in an experimental setting. Finally, the average age of participants was relatively high compared to other studies in the field.
**Psilocybin** (2mg in 10-mL saline, applied IV)	(Varley et al., 2020)	Open-label, within-subjects, Placebo-controlled.	N=15 healthy participants (2 females) were experienced psychedelic drug users Mean age = 32	NA	3T fMRI (BOLD & rsfMRI)	NA	**DMN modulation** Higuchi fractal dimension of BOLD time series was not significant for the DMN. **Supporting Model** REBUS Increased Higuchi fractal dimension was found in the Dorsal-Attentional network.	Limitations are small sample size and the Higuchi fractal dimension is not often used for BOLD signals as the number of samples in each time series is lower than EEG or MEG, decreasing the strength of these findings. Additionally, it is also likely that with psilocybin the time-series may be too short for the Higuchi fractal dimensions values to be reliable enough for replication. Furthermore, the parcellation values (1000 ROIs) may not be enough for a truly valid analysis of the fractal dimensionality of FC networks.
**Psilocybin** (0.17mg/kg, p.o.)	(Mason et al., 2020)	Double-blind, placebo-controlled, parallel-group design	N = 60 (25 female) healthy participants who had a history of psychedelic and substance use. Participants were excluded if they had used a psychedelic within 3 months of the study. Mean age = 23	5D-ASC scale and EDI were administered 360 minutes after drug administration.	7T fMRI (rsfMRI & MRS)	Administration of psilocybin was correlated with increased ratings of all sub-dimensions of 5D-ASC and the EDI.	**DMN modulation** Significantly less coactivation of the anterior and posterior DMN. Increased FC between the DMN and the FPN and SLN. Higher levels of glutamate in the mPFC, were associated with negatively experienced ego dissolution, and lower levels of glutamate in the mPFC was associated with positively experienced ego dissolution. **Additional Modulation** Widespread increases in between-network FC. **Supporting Model** REBUS	The increased BOLD sensitivity due to the high magnetic field may mean that geometric distortions become more prominent, thereby affecting the BOLD signal in inferior brain regions. Additionally, the scanning time was short from a test-retest perspective. The dose administered in this study was low-moderate compared to other trials, and this dose is also not capable of inducing a total ego dissolution.
**Psilocybin** (2 mg in 10-mL saline, applied IV)	(Carhart-Harris et al., 2013)	Within-subjects placebo-controlled study	N=15 healthy participants (2 females) were experienced psychedelic drug users. Mean age = 32	NA	3T fMRI (BOLD & rsfMRI)	NA	**DMN modulation** Significant increases in the FC between the anterior DMN and TPN’s such as the SLN, as well as the FPN, the AUD and dAN. **Additional modulation** Thalamic-DMN FC was nonsignificant **Supporting Model** REBUS	This was a new analysis of a previously published data set (Carhart-Harris et al., 2012), thus similar potential limitations and biases apply for the data collection process.
**Psilocybin** (2mg in 10mg of saline, administered IV)	(Muthukumaraswamy et al., 2013)	Within-subjects single-blind study	N = 15 healthy male participants. Mean age = 34.5	Upon 5 minutes of administration subjective experiences were deciphered using a 100-point VAS scale of 24 questions whilst in the MEG scanner. The 24 items consisted of questions like ‘I saw geometric patterns’, ‘the experience had a supernatural quality’ and ‘I felt a profound inner peace’.	MEG	17 out of 24 items were significantly higher in the psilocybin group compared to placebo, suggesting that psilocybin clearly altered participants subjective experience and was almost exclusively positive.	**DMN modulation** Decreased spectral power in key areas of the DMN such as the PCC. Specifically, decreases in the delta, theta, alpha, beta and low gamma frequency bands corresponded to the posterior regions. Decreased power and neuronal desynchronisation occurred in bilateral prefrontal cortices ranging from the alpha to gamma frequencies. **Supporting Model** REBUS	All participants had previous experience with psychedelic drugs and were all male, and were excluded if they have had an adverse side-effect to psychedelics resulting in decreased generalisability. The study was only single-blinded, rather than double-blinded. Finally, MEG artifacts were removed (caused by large muscle/head movements) at the pre-processing stage which may have been associated with specific aspects of the psychedelic experience.
**Psilocybin** (0.17mg/kg, p.o.)	(Mason et al., 2021)	Randomized, placebo-controlled, double-blind, parallel-group design	N=60 healthy participants (25 female) with previous experience with a psychedelic drug (but not within 3 months of the trial) Mean age = 23 Male to Female ratios were NA.	The 5D-ASC scale was administered 360 min after the drug and placebo intervention.	7T fMRI (MRS & rsfMRI)	Psilocybin increased ratings on all (sub)dimensions of the 5D-ASC, specifically ‘insight’ which was particularly pertinent to this study.	**DMN modulation** Significantly less coactivation was found within both the anterior and posterior DMN. Significant increases in between-network FC was observed for the DMN and the FPN and SLN. **Additional modulation** The FC within the FPN and SN was not significantly altered. **Supporting Model** REBUS	A limitation of the study is that behaviour measures taken outside of the scanner were correlated with resting-state connectivity.
**Psilocybin** (2mg in 10mg of saline, applied IV)	(Tagliazucchi et al., 2014)	Within-subjects placebo-controlled study.	N=15 healthy participants (2 females) were experienced, psychedelic drug users. Mean age = 32	NA	3T fMRI (BOLD & rsfMRI)	NA	**DMN modulation** Decreased low-frequency power and frequency scaling exponent (suggesting a poorly correlated signal) were observed in higher-order cognitive networks such as the DMN and this was juxtaposed with increased point processing rate. **Additional modulation** Increased amplitude of the BOLD signal was found within the hippocampus and ACC. **Supporting Model** REBUS	This was a new analysis of a previously published data set (Carhart-Harris et al. 2012), thus similar limitations and biases apply for the data collection process. Additionally, in this analysis, a limited number of regions were included in the definition of dynamical states. Consequently, it is possible that the change in brain dynamics is influenced/modulated by brain regions outside the scope of the analysis which involves known FC parameters to a pair of nodes.
**Psilocybin** (170 and 215 μg/kg p.o.)	(Kometer et al., 2015)	Double-blind, placebo-controlled study (within-subjects design)	N = 50 healthy subjects (22 females) Mean age = 25	The 5D-ASC was administered retrospectively (after all subjective effects of the treatment had dissipated) .	HD-EEG	Psilocybin significantly increased all sub-scaled of the 5D-ASC. Interesting there was a drug*dose*subscale interaction which led to a post-hoc analysis which showed that higher self-rated scores for hallucination and blissful states was dose-dependent.	**DMN modulation** Significant psilocybin-induced reduction in low-frequency bands (<20Hz) in the PCC and the retrosplenial cortex. Furthermore, there was a reduction in the source density in most frequency bands in regions such as the precuneus, ACC, cuneus and parahippocampal gyrus. Alpha 1 (8-10Hz) and partially alpha 2 (10.5-13Hz) reductions were found across areas in the posterior-parietal cortex and mainly isolated to the right hemisphere. **Supporting Model** REBUS	This paper relies on source localization and connectivity of deep structures (parahippocampal gyrus), which is highly contested as being possible with EEG.
**Psilocybin** (2mg in 10mL of saline)	(Roseman et al., 2014)	Within-subjects placebo-controlled study.	N=15 healthy participants (2 females) were experienced psychedelic drug users Mean age = 32	NA	3T fMRI (BOLD, rsfMRI & ASL)	NA	**DMN modulation** A significant increase in coupling between specific RSN such as: DMN-lFP, DMN-dAN, DMN2-ECN, DMN2-lFP, DMN2-dAN, DMN2-DAN2 and VisL-DMN. **Supporting Model** REBUS	This was a new analysis of a previously published data set (Carhart-Harris et al. 2012), thus similar limitations and biases apply for the data collection process. There was a significant correlation between head movement in the scanner and certain between network connectivity (i.e VisL-DMN).
**Psilocybin** *Open-label trial.* (10mg and 25 mg of oral psilocybin separated by a week.) *DB-RCT* Psilocybin arm 25 mg of oral psilocybin duplicated three weeks later. Between each dose, 3 weeks of daily placebo capsules were taken. Escitalopram arm Participants ingested 2 x 1 mg of psilocybin 3 weeks apart. Between psilocybin doses 10mg of daily escitalopram was orally ingested.	(Daws et al., 2022)		*Open-label trial* N = 19 patients (4 females) diagnosed with treatment-resistant depression. Mean age = 43 *DB-RCT* N = 59 patients (8 females) with major depressive disorder. Mean age = 44.5	In both experiments the Quick Inventory of Depressive Symptoms (QIDS) and the BDI-1A scores were used to assess depression severity in both studies. These measurements were taken at baseline, 1 week, 3 months and 6 months in the open trial and at 2,4 and 6 weeks in the DB-RCT.	3T fMRI (BOLD, rsfMRI)	Baseline scores of BDI were higher for the patients with treatment resistant depression then major depressive disorder. In the open-label trial psilocybin decreased median BDI scores at 1 week, 3 months and 6 months compared to baseline. In the DB-RCT both psilocybin and escitalopram decreased median BDI scores compared to baseline with the most pronounced decrease in the psilocybin condition.	**DMN modulation** *Open-label trial* DMN recruitment decreased and its between network integration with the ECN and SLN increased 1-day after psilocybin administration. Decreased brain modularity predicted improved clinical outcomes at 6 months post treatment. *DB-RCT* Brain network modularity was significantly reduced at the primary end point, 3 weeks after psilocybin therapy (no differences were found for escitalopram). This decrease in modularity was associated with positive treatment outcomes. **Additional modulation** Post-psilocybin changes in the dynamic flexibility of various brain networks were correlated with changes in depressive symptomatology. **Supporting Model** REBUS	**Limitations** There was a much larger proportion of males to females in the study and fMRI scans were conducted while eyes were closed so it is possible that participants may have fallen asleep for periods of time. Repeated scanning sessions would have also been superior to single scanning recordings. Psilocybin did not outperform citalopram in QIDS scores in the latter trial, and QIDS was the primary outcome of the original trials. However, in the re-analysis the Beck’s Depression Inventory was used as the primary analysis. Furthermore, testing significance with a one-tailed t-test (rather than two tailed) despite an a-priori prediction has been challenged as has the conducting of a post-hoc analysis (between the two treatments) without there being a significant interaction.
**Ayahuasca** (1mL/kg, 0.36 mg/mL of N,N-DMT, 1.86 mg/mL of harmine, 0.24 mg/mL of harmaline, and 1.20 mg/mL of tetrahydroharmine)	(Pasquini et al., 2020)	Randomized placebo-controlled trial	N = 43 physically and mentally healthy, who were ayahuasca naïve (20 males). Mean age = 31	Acute psychedelic experience was quantified via the Hallucinogenic Rating Scale (HRS). This was administered approximately 4 hours after ayahuasca and placebo ingestion.	1.5T fMRI (BOLD & rsfMRI)	Ayahuasca increased all subscales of the HRS which all survived multiple correction testing with the exception of the volitional subscale.	**DMN modulation** Significant decreases in FC within the PCC and increased FC within the ACC. There is an increase in the FC between the DMN and the SLN, VN and the SMN. **Supporting Model** REBUS	A significant limitation is the application of liberal voxel-wise statistical thresholds which can increase the likelihood of false-positive (type 1 error) results. The study garnered a low statistical power due to the sample size and strength of the MRI scanner (1.5T).
**Ayahuasca** (2.2 mL/kg, 0.8mg/ml of DMT and 0.21 mg/ml of harmine)	(Palhano-Fontes et al., 2015)	Open-label, Cohort study.	N = 10 participants (5 male) who were mentally healthy and had at least 5 years of regular (twice a month) ayahuasca use. Mean age = 29	Subjective effects caused by Ayahuasca were measured via the BPRS and the YMRS at baseline, 40 minutes, 80 minutes and 200 minutes.	1.5T fMRI (BOLD & rsfMRI)	5 out of the 11 items in the YMRS were statistically significant (at 80 min). These were elevated mood, speech, increase motor activity-energy, speech, language-thought disorder and content compared to baseline. The results from the BPRS were not presented.	A reduction in the BOLD signal of the DMN regions such as mPFC, ACC, PCC, IPL which is primarily driven by a reduction in the PCC/Precuneus seed. No significant difference in DMN-TPN orthogonality was found after ayahuasca consumption. **Supporting Model** REBUS	The study was limited to experienced users, limiting generalisability. The fixed effects analysis used, further limits the generalisability of the findings. No placebo group was present. The acquisition of fMRI data was not controlled for order effects. The sample of 10 participants is comparatively low to similar studies. The 1.5T fMRI is known to have decreased spatial resolution when compared to an MRI scanner with a higher magnetic field.
**Ayahuasca** (0.3 mg/mL DMT, 0.86 mg/mL harmine, 0.17 mg/mL tetrahydroharmine, and 0.04 mg/mL harmaline. Average consumption was 148 mL.)	(Sampedro et al., 2017)	Open-label, uncontrolled study	N = 16 healthy volunteers (6 females) with prior experience with ayahuasca. Mean age = 39	Participants filled out two questioners in the post-acute stage of the intervention. For enduring effects these questioners were then administered at a two month follow up. These questionaries were the HRS and a mindfulness questionnaire. The mindfulness questionnaire was a Spanish adaptation of the FFMQ, the Experiences Questionnaire and the short version of the Self-Compassion questionnaire. The mindfulness questionnaire was also administered at baseline.	3T fMRI (BOLD & rsfMRI)	At the post-acute stage significant differences from baseline were observed for the FFMQ non-judging and nonreacting subscale.	**DMN modulation** Glutamate levels in the PCC were lower compared to baseline (post-acute phase). Increased coupling from the PCC to the ACC, and visual areas were found. **Additional modulation** The superior rostral ACC seed was found to have increased coupling with subcortical regions such as the parahippocampal, hippocampal and amygdala. **Supporting Model** REBUS	There was no control/placebo group and all participants were experienced ayahuasca users (who showed high levels of baseline mindfulness). The study only looked at sub-acute effects and cannot be interpreted to infer the same persistent changes.
**Ayahuasca**	(Bouso et al., 2015)	Cross-sectional study.	N = 44 psychologically healthy participants (12 male) who had taken ayahuasca an average of 123 times, with minimal exposure to other drugs. Mean age = 41	Personality was assessed at using a Spanish version of the TCI-R questionnaire.	3T fMRI (BOLD)	Ayahuasca had a significant difference in harm avoidance (specifically the sub-item of anticipatory worry) and self-transcendence compared to controls.	**DMN Modulation** Cortical thinning was observed in the precuneus, the MFG, SFG and the PCC. Conversely, there was cortical thickening found in the ACC and precentral gyrus. **Supporting Model** REBUS	Due to the cross-sectional nature of the study, causality could not be inferred/established. The participants were also limited to long term users and what level of ayahuasca use (i.e., a certain threshold) to illicit the corresponding change in brain structure was not determined.
**LSD** (100 μg, p.o.)	(Müller et al., 2018)	Double-blind, randomised, cross-over study	N = 20 healthy (10 male) participants. Only 2 participants had previous psychedelic use and both were only on a single occasion. Mean age = 32	Subjective effects were measured three hours after the ingestion of psilocybin or placebo, using the 5D-ASC scale.	3T fMRI (BOLD)	Ratings in all major dimensions of the 5D-ASC were significantly increased for the LSD group compared to the placebo-control	**DMN modulation** Significantly decreased coactivation within the medial-posterior DMN was observed. No significant correlation was found between DMN integrity and ego dissolution measures. Widespread increases in between network FC were observed (i.e increased FC between the DMN and the SLN, VN and SMN). **Additional modulation** Decreased coactivation also occurred parts of the VN and sensorimotor network. Additionally, there was Increased FC between key hubs such as the precuneus, ACC, striatum and RSN’s for the LSD condition compared to placebo. **Supporting Model** None	Measurements of known FC confounders like increased heart rate and blood pressure (which LSD is known to alter), were only recorded at one-time point before the fMRI scan and a nuisance regression that continuously monitored these parameters may have been optimal.
**LSD** (75µg, IV injected)	(Lebedev et al., 2016)	Placebo controlled counter-balanced design.	N = 20 healthy volunteers (four females). All participants were required to have had previously used a classical psychedelic (Mean LSD use = 14). Mean age = 31	The standard 240-item revised NEO-PI-R, was administered twice to assess (possible) personality changes invoked by LSD. The NEO-PI-R was administered at baseline and 2-weeks after the LSD or placebo intervention. A VAS was completed inside the scanner and immediately after the scanning by the participants. A key facet of experience that the VAS questions assessed was ego-dissolution.	3T fMRI (BOLD, & rsfMRI)	In-scanner reports of ‘ego dissolution’ were significantly positively correlated with the ‘mystical’ quality of the experience.	**DMN modulation** Increased cortical entropy and significant increases were observed in the DMN and precuneus. **Additional modulation** Cortical entropy was a predictor of changes in the personality trait openness. **Supporting Model** REBUS	One of the MRI scanning conditions listened to music and the order of this group was not counter-balanced which obscures its specific influence from pharmacodynamics. Furthermore, music may also have facilitated the entropic effects of LSD by inducing a meditative like states in participants, leading to greater effect sizes and significance.
**LSD** (75 µcg (IV) in 10 mL saline)	(Speth et al., 2016)	Placebo-controlled crossover study.	N = 20 healthy volunteers (four females). All participants were required to have had previously used a classical psychedelic (Mean LSD use = 14). Mean age = 31	The size of the subjective effects of LSD were quantified and assessed by a VAS with 20 increments.	3T fMRI (BOLD & rsfMRI)	The subjective effect of LSD was first detected around 10 minutes after LSD infusion, and peaked around 120 minutes after LSD administration. After 7-8 hours after IV LSD, subjective effects subsided.	**DMN modulation**DMN disintegration predicted fewer cognitive agencies related to mental space for the past (i.e less mind-wandering to past events). **Supporting Model** REBUS	A small sample size (n = 15, 5 were excluded) were used for the fMRI scan which can lead to false positives and unreliable results. It is also possible that reduced mentation to the past was not a unique feature of LSD but rather a motif shared by many other intoxicants. Finally, one of the key findings, that DMN disintegration predicts less mentation of the past was not statically significant (p = 0.54), yet the authors seem to think that the mild correlation strength (r = 0.51), is sufficient to still conclude that DMN disintegration is correlated with memories of the past.
**LSD** (75 µg of LSD, administered IV via a 10ml solution)	(Jobst et al., 2021)	Placebo-controlled crossover study	N = 20 healthy volunteers (four females). All participants were required to have had previously used a classical psychedelic (Mean LSD use = 14). Mean age = 31	Not Applicable	3T fMRI (BOLD & rsfMRI)	Not Applicable	**DMN modulation** A significant difference in the PILI of the DMN for the LSD condition compared to placebo. **Additional modulation** A significant difference in the PILI of the limbic RSN for the LSD condition compared to placebo. Increased variability in brain states across all brain regions was also observed. **Supporting Model** REBUS	The model of analysis used contained some limitations such as homogeneity. For instance, there was a presupposition that all brain regions have the same intrinsic neural dynamics (alpha = 0), thus the differences in the dynamics of this model were a result of varying effective connectivity. However, it is not obvious that intrinsic neural dynamics are constant in the brain and most likely vary between networks, brain structures, and cell types. Furthermore, since the model was constructed upon BOLD signals the frequency range was limited to slow frequencies.
**LSD** (13 μg and 26 μg p.o.)	(Murray et al., 2022)	Double-blind, placebo-controlled study	N = 22 (8 female)Mean age = 25 Participants were healthy young adults with prior use of psychedelics (unless they experienced an adverse reaction).	Subjective mood states were recorded before drug intake and in 60-min intervals after drug administration. These measures were the DEQ, the ARCI, and the POMS. Furthermore, at the end of each session participants were required to complete a drug-identification and 5D-ASC questionnaire.	EEG	LSD significantly increased ratings of ‘Feel High’, ‘Like Drug’ and ‘Want More’ on the DEQ. Likewise, LSD (compared to placebo) significantly increased Elation, Anxiety and Positive Mood on the POMS. Finally, on the ACRI LSD had amphetamine-like effects, euphoric effects and increased energy and intellectual efficiency. The peak subjective experiences occurred 2-3 hours upon LSD ingestion. The end of session questionnaire revealed that most participants correctly identified whether they received a ‘placebo’ or ‘hallucinogen’ at the 26 μg session. For the 13 μg LSD session guesses were less accurate. The 26 μg group was the only group to significantly alter scores on the 5D-ASC.	**DMN modulation** LSD reduced oscillatory amplitude across three major hubs of the DMN (mPFC, PCC, bTPC) for five frequency bands (delta [1-4 Hz], theta [4-8 Hz), alpha [8-13 Hz], beta [13-30 Hz] and gamma [30-80Hz]). The effects were most pronounced for electrodes placed on the PCC. **Supporting model** REBUS	An important limitation of the data extraction and analysis is the fact that a dipole or distributed source detection of intracranial spectral density was not included. Therefore, this study is prone to the inverse problem of inferring the source of scalp recorded signals from deeper brain regions.
**LSD** (75 µg of LSD, administered IV via a 10ml solution)	(Varley et al., 2020)	Single-blind, placebo-controlled balanced-order design	N = 20 healthy volunteers (four females). All participants were required to have had previously used a classical psychedelic (Mean LSD use = 14). Mean age = 31	Not Applicable	3T fMRI (BOLD & rsfMRI)	Not Applicable	**DMN modulation** Higuchi fractal dimension was found to be insignificant for the DMN. Additional modulation **Additional modulation** Significant increase in fractal dimensions in the visual network, dorsal-attention network, and fronto-parietal network. **Supporting model** REBUS	The same limitations and biases as Varley et al. (2020) (psilocybin), apply, which have been outlined above.
**LSD** (75 µg of LSD, administered IV via a 10ml solution)	(Carhart-Harris et al., 2016)	Single-blind, placebo-controlled balanced-order design	N = 15Mean age = 31 Participants all had previous psychedelics use (except within 6 weeks of the first scanning day) and were healthy individuals.	An 11-factor ASC questionnaire was completed at the end of each scanning day. An additional questionnaire was administered directly after MRI and MEG scanning and consisted of VAS-style ratings for 21 items.	3T fMRI (BOLD & ASL) and MEG	All of the items on the ASC questionnaire were significantly increased for LSD compared to placebo with the exception of anxiety. Upon MRI scanning, participants rated 18/21 items significantly higher than placebo. The three items which showed no significant difference where ‘I felt afraid’, ‘I feared losing control of my mind; and ‘I felt suspicious and paranoid’ The results were similar for the MEG group where 15/21 Items were significantly different as a results of LSD administration compared to placebo. A control item for both scans ‘I felt entirely normal’ was rated higher for the placebo group.	**DMN modulation** There was decreased DMN integrity and CBF and this correlated with measures of ego dissolution. Decreased segregation between the DMN and salience network was observed, however, this was not significantly correlated with ego dissolution. **Supporting model** REBUS	The indirect measure of neural activity via CBF, should be approached with caution as this measure has poor temporal resolution and is prone to the confounder of neurovascular coupling as a result of the drug. Additionally, the experimental protocol may have been strenuous for participants with the different scan types of ASL, BOLD and MEG, where fatigue may occur and confound findings. This could be overcome by a simultaneous EEG-fMRI analysis.
**LSD** (100 µg p.o)	(Preller et al., 2018)	Double-blind, randomized, cross-over study	24 healthy participants (19 males) Mean age = 25	5D-ASC	3T fMRI (rsfMRI)	LSD significantly increased mean scores across scales on the 5D-ASC with a peak effect at 180 minutes after administration	**DMN modulation** GBC was found within higher order associative brain regions. For instance, reduced GBC was found within the mPFC, a key DMN brain region. **Additional modulation** Increased GBC across sensory and somatomotor functional networks was observed. Interestingly the hyper-and hypo- connectivity motifs across the brain were significantly correlated and driven primarily by 5-HT_2A_ and possibly 5-HT_7_ antagonism. Changes in connectivity patterns within the somatosensory network and not the DMN were associated with subjective experiences. **Supporting Model** REBUS and CSTC	Although a strength of this study is the removal of global signal artifacts, it is possible that such an analysis can also remove pharmacologically induced brain signal alterations. Furthermore, there was more than three times as many males than females in the cohort.

**Abbreviations**: SLN (salience network), CBF (cerebral blood flow), VN (visual network), AUD (auditory network), RSN (resting-state network), TPN (task-positive network), FPN (frontoparietal network), PILI (Perturbational Integration Latency Index), bTPC (bilateral temporoparietal cortex), IPL (inferior parietal lobule), dAN (dorsal attention network), VisL (visual-Lateral), DMN2 (Hybrid of anterior DMN and Executive Control Network), ECN (executive control network), lFP (left frontoparietal network), ASL (arterial spin labelling), DTI (Diffusion Tensor Imaging), IV (intravenously), BOLD (Blood Oxygen Level Dependent) HD (High Density) rs (resting state), NA (not available), MRS (Magnetic Resonance Spectroscopy), QIDS-SR16 (Quick Inventory of Depressive Symptoms), 11D-ASC (11- Dimension Altered States of Consciousness), MEQ (Mystical Experiences Questionnaire) and EDI (Ego-Dissolution Inventory), Persisting Effects Questionnaire (PEQ), NEO PI-R (NEO Personality Inventory-Revised), MAAS (Mindfulness Attention Awareness Scale), PANAS-X (the Positive and Negative Affect Scale), POMS (The Profile of Mood States), DPES (The Dispositional Positive Emotions Scale), DASS (Depression Anxiety Stress Scale), GBC (Global Brain Connectivity), MEG (magnetoencephalography), (DEQ) Drug Effects Questionnaire, ACRI (Addiction Research Centre Inventory), bTPC (bilateral temporoparietal cortex), Temperament and Character Inventory-Revised (TCI-R), MFG (medial frontal gyrus), SFG (superior frontal gyrus), Five Facet Mindfulness Questionnaire (FFMQ), Young Mania rating Scale (YMRS), IPL (Inferior parietal lobule), BPRS (Brief Psychiatric Rating Scale), DB-RCT (Double-blind randomised control trial).

Alterations in the FC signatures of the DMN, consisting of intra-connectivity changes (FC alterations within the DMN brain regions) and inter-connectivity changes (changes in FC parameters between brain regions within the DMN and brain regions within other cognitive networks), have been implicated in a variety of complex cognitive functions such as theory of mind, self-referencing, memory, and rumination (Andrews-Hanna, 2012; Raichle, 2015). However, the underlying mechanisms are not fully understood (Smallwood et al., 2021). Additionally, altered DMN function has been implicated in a range of neuropsychiatric and neurodegenerative conditions such as depression, attention deficit hyperactivity disorder, schizophrenia, anxiety, and post-traumatic stress disorder, Alzheimer’s disease, and aging in general (Mohan et al., 2016; Zhang and Volkow, 2019). As such, altered DMN connectivity in specific clinical populations or groups consuming therapies that impact functional brain activity (e.g., psychedelics) appears to offer a window into the variability of complex human functioning that can also be modulated by psychedelic treatment.

The “mind-manifesting” properties of psychedelics illuminated by figures like Aldous Huxley, Timothy Leary, and Albert Hofmann created a surge of trials during the 1960s, which highlighted psychedelics as potential therapeutic agents for a range of mental health and substance use disorders (Carhart-Harris, 2019). In particular, lysergic acid diethylamide (LSD) was on the market as Delysid/Sandoz in the 1950s and 1960s, prescribed for the treatment of “psychoneuroses, psychoses” as well as other neuropsychiatric disorders, always to be administered in a controlled setting (psychiatric clinic or hospital) by properly trained health professionals. The use of LSD and all other psychedelics in clinical or preclinical research was severely constrained for political reasons in the 1970s, although LSD continued to be prescribed by psychiatrists outside the United States well into the 1990s. Based on the promising yet preliminary findings with LSD and other drugs such as psilocybin, N,N-dimethyltryptamine (DMT), ayahuasca, or mescaline, but also 3,4-methylenedioxymethamphetamine (MDMA) and ketamine, psychedelics are regaining scientific attention, especially in the field of clinical neuroscience. As a result, a number of more recent studies have begun to elucidate how psychedelics modulate the DMN (Carhart-Harris et al., 2012, 2016; Bouso et al., 2015; Palhano-Fontes et al., 2015; Thomas et al., 2017; Millière et al., 2018; Müller et al., 2018; Kelly et al., 2019; Smigielski et al., 2019; Mason et al., 2020; Mertens et al., 2020; Daws et al., 2022; de Gregorio et al., 2021; Grandjean et al., 2021).

Psychedelics often induce meaningful and mystical experiences that have been associated with increased measures of brain entropy (Carhart-Harris, 2018). Entropy is a measure of the uncertainty of the system, and regarding the brain, it is associated with functional disorder, unpredictability, and flexibility, which may lead to an enhanced array of dynamic brain states (Tagliazucchi et al., 2014; Atasoy et al., 2017; Carhart-Harris, 2018). Yaden and Griffiths (2020) argue that a considerable variance in the therapeutic outcome associated with psychedelics can be explained by the altered state of consciousness that psychedelics produce. Furthermore, one of the most ubiquitous and transformative components of the psychedelic experience is the feeling of ego dissolution, a relaxation of subject-object distinctions during which the borders and constraints of the self seem to dissolve (Letheby and Gerrans, 2017; Mason et al., 2020). Feelings of ego dissolution also appear to be a central component of the unitive experience (a sub-component of the mystical states), which is an experience of perceived union with nature or a higher power or an all-pervading sense of oneness (Nour et al., 2016). It has been hypothesized that ego dissolution can be therapeutic because an individual’s cognitive attributions and affect are viewed with a greater distance and objectivity (Letheby and Gerrans, 2017; Mason et al., 2020). Psychedelic-induced ego dissolution may be precipitated via Bayesian belief updating wherein reduced precision of previously held beliefs lends to revision of the self and world, as hypothesized by Stolicker and colleagues (2022). This has parallels with the practice of meditation, which is also associated with decreased activity of the DMN and alterations to precision-weighting of beliefs and attention (Brewer et al., 2011; Palhano-Fontes et al., 2015; Letheby and Gerrans, 2017; Millière et al., 2018). Functional magnetic resonance imaging (fMRI) studies involving psychedelics show that the modulation of the DMN acutely decreases connectivity and blood flow within nodes of this network (Table 2). These changes are paralleled by magneto/electroencephalography studies showing neuronal desynchronization of alpha power (itself previously shown to be highly correlated with DMN connectivity), at times specifically located in the PCC (one of the main hubs of the DMN), which has been tentatively hypothesized to result in a mind that is less constrained, more flexible, and less self-referential and egoic (Muthukumaraswamy et al., 2013; Letheby and Gerrans, 2017; Nour and Carhart-Harris, 2017; Carhart-Harris, 2018; Mason et al., 2020).

Although the DMN is a RSN that is featured in a vast array of psychedelic trials, there is still some ambiguity as to what changes/modulation to this network mean and what motifs and differences are seen across the literature regarding this network. Therefore, this systematic review seeks to explore the following aims: (1) to critically evaluate the impact of classic psychedelics on the DMN; (2) to evaluate the evidence regarding DMN modulation and resultant psychotherapeutic effects; (3) to identify challenges and limitations associated with the current literature surrounding DMN modulation by psychedelics; and (4) to provide a discussion surrounding future research and the potential future utility of DMN modulation–focused psychedelic therapies. This review focuses on the classical psychedelics: LSD, psilocybin, DMT, mescaline, and ayahuasca (which contains DMT and harmala alkaloids), complementing previous reviews of these agents (Carhart-Harris and Friston, 2019; Aday et al., 2020; Inserra et al., 2021). To this end we conducted a literature review via Scopus and PubMed databases on August 28, 2021, up to November 14, 2021.

## METHODS

This systematic review followed the PRISMA statement (Moher et al., 2015) for transparent and comprehensive reporting. The review was not registered, and a review protocol was not prepared.

### Search Strategy

We conducted an electronic database search of PubMed and Scopus from inception to April 2022. We structured our search according to the PICO framework (Schardt et al., 2007) using search terms related to the DMN, along with the names of the classical psychedelics, to return all potentially eligible studies. The search string was: default mode network OR DMN OR neuro circuitry AND ayahuasca OR DMT OR dimethyltryptamine OR psilocybin OR psilocin OR LSD OR Lysergic acid diethylamide OR mescaline OR peyote OR psychedelic. A search of Google Scholar was conducted to identify any additional relevant articles.

### Eligibility Criteria

**Figure 2.**
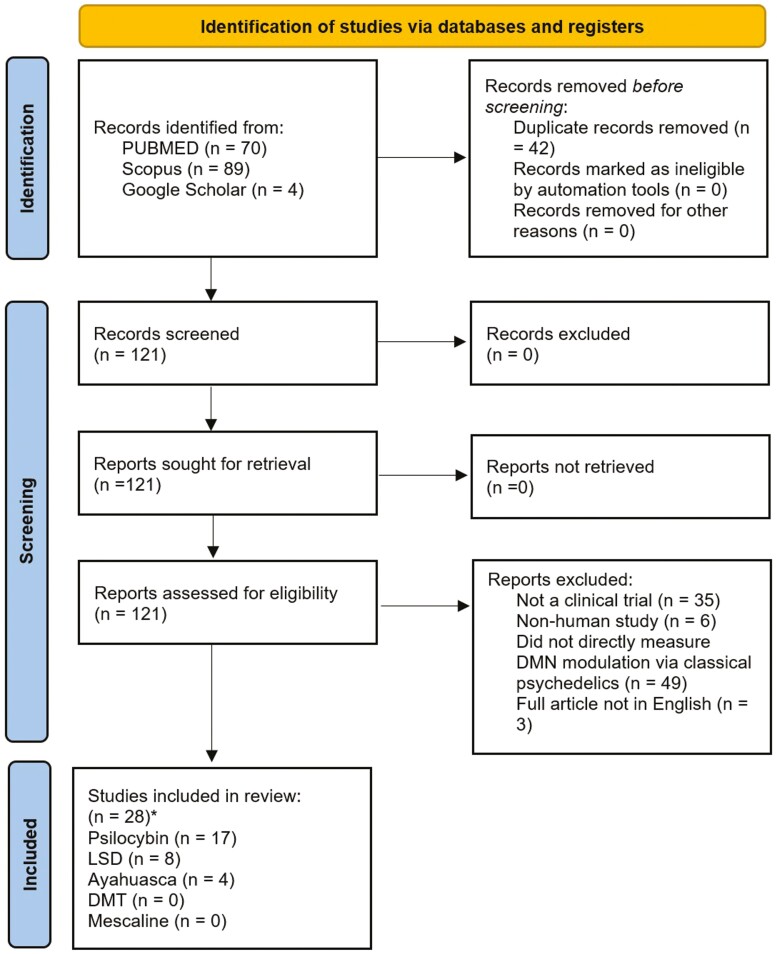
PRISMA flow diagram (Page et al., 2021) for study selection. Although 28 papers were included, 1 paper (Varley et al., 2020) included 1 analysis on psilocybin and 1 on LSD.

Articles were screened by 2 reviewers (J.G., S.R.). Disagreements were resolved through discussion until consensus was reached (with J.S. arbitrating if required). We included only human clinical trials (rodent studies were excluded) assessing DMN modulation by the classical psychedelics. Papers that performed novel analyses on previously published datasets were also included [i.e., Carhart-Harris et al., (2013) and Varley et al., (2020)]. Only English language papers were eligible, and there was no psychedelic dose requirement and no requirement on the length of treatment or on the statistical methods used. All clinical diagnoses and healthy controls were included. The full article screening and selection process is detailed in Figure 2.

## RESULTS

### Results Overview

The initial database search was performed on 28th of August 2021 and a second database search was performed on the 20th of April 2022. The search returned 163 results which were reduced to 119 after the duplicates were removed. A further 95 articles were removed due to ineligibility. This left 28 articles that were included in the study (Figure 2).

**Figure 3.**
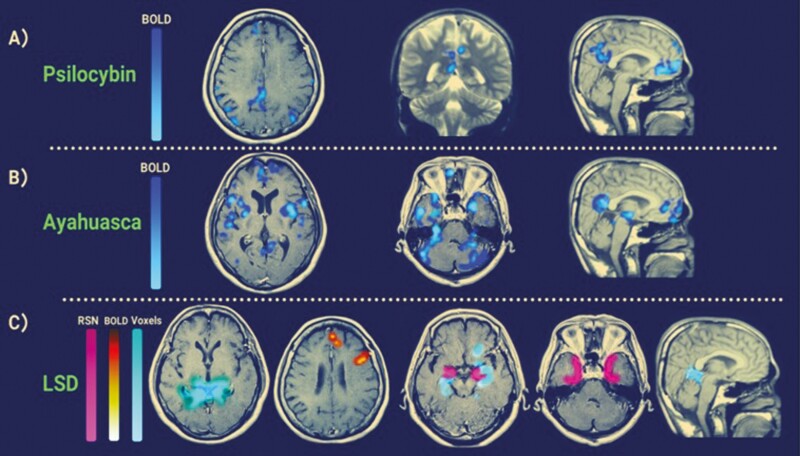
fMRI activity patterns of the various psychedelics on regions of the DMN. (A) Blue BOLD signals represent significant brain deactivations after psilocybin compared with placebo (adapted from Carhart-Harris et al., (2016)). (B) Significant BOLD decreases of the DMN after the ingestion of Ayahuasca (adapted from Palhano-Fontes et al., (2015)). (C) Between-group differences (LSD vs placebo) in the FC between a key DMN node (bilateral hippocampal seed) and the rest of the brain. Orange represents increases in FC between the seed at the parahippocampus, and cyan/blue signifies decreases. Pink is a mask of the parahippocampal gyrus [adapted from Carhart-Harris et al., (2016)]. This figure is used to help visually compare and contrast the heterogeneity of activity patterns between 3 foundational studies, but it should be noted that other neuroimaging studies exist that are not included. Blood-oxygen-level-dependent (BOLD), Default mode network (DMN), Functional connectivity (FC).

A total of 17 psilocybin studies were assessed, twelve using fMRI (2 of 12 using 7 Tesla fMRI, remaining using 3 Tesla) and two using Electroencephalogram (EEG) or Magnetoencephalogram (MEG). The average age of subjects across these studies was 34.6 years with a standard deviation (SD) of 8.8 years. The average number of participants across the studies was 26 with a SD of 19. Out of the 17 studies, 13 had a placebo control group. Four Ayahuasca studies were assessed, with all four using fMRI (two using 1.5 Tesla and two, 3 Tesla). The average age of subjects across these studies was 35 years with a SD of 17.8 years, and the average number of participants was 28 with a SD of 5.9 years. Only one of these studies had a placebo control group. Eight LSD studies were assessed, with seven using (3 Tesla) fMRI, one using EEG and one using MEG. The average age across these studies was 29.6 years with a SD of 2.8, and the average number of participants was 20, and the SD was 6.4 participants. All studies had a placebo control group. No published research papers assessing how mescaline and DMT modulates the DMN in humans were found. Table 2 is a summary of the findings and study characteristics for the research articles included in this systematic review. Figure 3 shows the specific DMN brain regions detected via fMRI that are modulated by psychedelics.

## DISCUSSION

### Synthesis of Data

Based on the evidence (see Table 2), there are clear associations between a psychedelic’s ability to reduce the functional connectivity within the DMN (and increase its connectivity to other networks), altered states of consciousness, and therapeutic outcomes (Letheby and Gerrans, 2017; Müller et al., 2018; Smigielski et al., 2019; dos Santos and Hallak, 2021; Mason et al., 2021; Daws et al., 2022). At this stage, however, it is difficult to ascertain the direction of causality.

A recent review from Aleksandrova and Phillips (Aleksandrova and Phillips, 2021) also revealed that the classical psychedelics can induce marked neuronal structural and functional changes via neurotrophic signaling and neuroplasticity. These psychoplastic changes are most likely mediated by brain-derived neurotrophic factor and mammalian target of rapamycin (Inserra et al., 2021). Furthermore, Martin and Nichols (2017) showed that psychedelics have the potential to alter gene expression and immunomodulatory mechanisms. These effects are proposed to rewire pathological cortical networks (possibly by reducing neuronal inflammation, a burgeoning hypothesis of addiction, depression and neurodegeneration), thereby explaining how psychedelics may neurobiologically induce positive long-term health outcomes. Thus, it is challenging to delineate how much of the benefit in psychedelic therapy is derived from neuroplastic changes on the cellular level or on the network level (DMN modulation) and the degree to which the two are linked. This is further confounded by the fact that studies use varying dosages of psychedelics are not all placebo controlled and often investigate participants who are not drug naïve.

Depression is often characterized by excessive activity in the mPFC, and there appears to be an inverse correlation between depressive symptomatology and the degree of 5-HT_2A_ receptor stimulation in this region (Morris, 2008; Nichols, 2016; Carhart-Harris, 2019). Psilocybin can acutely increase anxiety, which may be mediated by glutamate hyperfrontality; however, long-term reductions in anxiety could occur due to 5-HT_2A_ receptor downregulation in this area (Mason et al., 2020). Another hypothesis is that psilocybin, and the classical psychedelics more broadly, have a significant affinity for and agonize the 5-HT_1A_ receptors. These are the primary inhibitory serotonergic receptors and may be responsible for the decreased blood oxygen level–dependent (BOLD) coactivation often seen in the DMN and other brain regions on the administration of psychedelics. On the macro-scale, connectivity between the parahippocampal cortex and prefrontal regions was a reliable predictor of depressive symptoms 5 weeks after psilocybin treatment (Carhart-Harris et al., 2018).

A review by Thomas et al. (2017) concluded that based on the totality of evidence at that time (7 clinical trials), psilocybin, by disrupting the hyperconnectivity in the DMN of psychiatric conditions, may be a novel treatment for a range of neuropsychiatric conditions. A recent analysis by Daws et al. (2022) found that psilocybin therapy reduced depressive symptoms for up to 6 weeks post intervention and that this effect was likely dependent on increased brain network integration. Interestingly, this group also found that increased within-DMN connectivity and reduced between-network DMN connectivity (specifically with the executive and salience network) were correlated with baseline depressive severity, which aligns with the previous findings of other researchers (Hamilton et al., 2011; Lydon-Staley et al., 2019; Feurer et al., 2021).

The literature reviewed clearly indicates that classical psychedelics acutely decrease functional connectivity within the DMN and increase between-network connectivity (see Table 2) (Roseman et al., 2014; Lebedev et al., 2015; Palhano-Fontes et al., 2015; Carhart-Harris et al., 2016; Nichols, 2016; Tagliazucchi et al., 2016; Atasoy et al., 2017; Müller et al., 2018; Mason et al., 2020; Mertens et al., 2020; de Gregorio et al., 2021; Grandjean et al., 2021; Daws et al., 2022; Madsen et al., 2021). Thus, during the psychedelic experience, there seems to be a unique shift in neural connectivity, reflecting a shift from a more modular, segregated brain to a more interconnected global network (Palhano-Fontes et al., 2015; Smigielski et al., 2019). This decrease in between-network segregation seems to be somewhat specific to the classical psychedelics, although this decreased segregation is also seen in ketamine (Bonhomme et al., 2016) and salvinorin A (Doss et al., 2020). It is not seen with selective serotonin reuptake inhibitor antidepressants (Klaassens et al., 2015; Daws et al., 2022), stimulants (Konova et al., 2015), sedatives (Stamatakis et al., 2010), or MDMA (Roseman et al., 2014). This increase in inter-network FC can be interpreted via a dynamical systems theory approach [see Roseman et al., (2014) for more details]. For instance, RSNs are canonical networks of brain activity and when reflected graphically, the valleys in this 2D plane are indications of the metastable “sub-states” of these RSNs. Thus, deeper valleys imply a prolonged sub-state with greater rigidity, whereas shallower valleys indicate less stable and more flexible sub-states. The increase in FC between the DMN and other RSNs seems to align with the latter graphical representation (i.e., shallow troughs), which may indicate that the brain/mind can access a greater dynamic repertoire of metastable sub-states. This more flexible brain state seems to persist in the recovered state of patients with major depressive disorder (MDD) treated with psilocybin (Daws et al., 2022).

Psychedelics may in part disrupt the DMN because they promote a cognitively intensive experience where one is dealing with the “task” of one’s subconscious and grappling with the deep metaphysical and personal questions that generally arise during the experience (Shanon, 2002). This aligns with the definition of the DMN, which is anticorrelated with task-based activities. Although mind-wandering is associated with increased DMN activity, awareness of mind-wandering—as seen in meditation—decreases the resting FC of the DMN (Brewer et al., 2011; Palhano-Fontes et al., 2015). This is a plausible mechanism by which ayahuasca, as well as psilocybin and LSD, also decrease activity within the DMN because the experience is characterized by a hyper-awareness of one’s internal dispositions and attributions (Brewer et al., 2011; Preller and Vollenweider, 2016; Millière et al., 2018). Furthermore, it could be argued that hyper-awareness of one’s mind and internal cognition is a feature of a brain that has increasing entropy and is approaching criticality (Carhart-Harris et al., 2012, 2016; Carhart-Harris, 2018; Millière et al., 2018), although this needs to be further examined. It is also clear that various individuals will respond differently to distinct treatments because their baseline levels of awareness will differ. Another plausible mechanism of reduced coactivation within the DMN is that psychedelics produce less mentation to the past, as seen with LSD, but whether this effect is unique to psychedelics is unclear (Speth et al., 2016).

### Models of Psychedelic Action

It is important to note that the “relaxed beliefs under psychedelics” (REBUS) model is 1 of 3 prominent models of the biological mechanisms underpinning psychedelics’ mode of action, and each model exhibits unique strengths and weaknesses. The REBUS model has been the primary focus of this article (refer to Table 2, where 24 of the 28 papers were most closely aligned with the REBUS model) because it included DMN activity and modulation as a central axiom (REBUS is based on the brain’s hierarchical organization with DMN at the top of this hierarchy). The two other models suggest mechanisms that are relatively independent of DMN modulation (Doss et al., 2022).

#### Relaxed Beliefs Under Psychedelics and Brain Entropy

It has been hypothesized that the underlying neurobiology of DMN perturbation and its downstream effects is caused by entropy (Carhart-Harris, 2018). Psychedelics exert their hallucinogenic effects primarily by activating the 5-HT_2A_ receptor, resulting in increased 5-HT release, enhancing the excitability of layer V pyramidal cells in the cortex and hence leading to increased glutamate in the neocortex (Nichols, 2016; Carhart-Harris, 2019; Mason et al., 2020). In the cortex 5-HT2A receptor activation can lead to asynchronous glutamate release resulting in desynchronized ensembles of neurons (Nichols, 2016; Riba et al., 2002; Tagliazucchi et al., 2014). Subsequently, the brain becomes more desynchronized resulting in a loss of oscillatory power, aligning with the brain entropy hypothesis (Aghajanian and Marek, 1999; Tagliazucchi et al., 2014; Carhart-Harris, 2018).

Brain entropy is characterized by increased randomness, unpredictability, and disorderliness (with respect to neuronal firing), which in turn disrupts top-down, goal-oriented cognitive processing (Carhart-Harris, 2018, 2019; Kelly et al., 2019; Varley et al., 2020). It is proposed that this disruption in top-down processing may facilitate increased neural and cognitive flexibility (Doss et al., 2021), which is a viable explanation for the mechanistic underpinnings of the therapeutic effect of psychedelics (Gallimore, 2015; Mason et al., 2021; Daws et al., 2022). The REBUS model postulates that psychedelics serve to relax the precision-weighting of previous beliefs while freeing up and increasing the information flow from bottom-up information processing (Carhart-Harris and Friston, 2019). This process can enable the revision of pathologically overweighted priors, which affects the functional systems of the brain such as those related to the self (Ho et al., 2020).

Evidence from Barrett et al. (2020a) and Daws et al. (2022) may support this model and the notion of a “resetting mechanism” because the acute disruption in top-down cognitive control showed enduring benefits (up to 1 month) in top-down control of emotion, leading to less negative affect and increased positive affect after psilocybin administration. Anatomical connectivity can be conceived as a Bayesian prior on FC, and under anesthetic, external information is minimally processed and integrated, which means that there is a strong association between the FC and structurally encoded priors. However, under classical psychedelics, the brain is less constrained by pre-existing structural priors and therefore these pre-existing priors have less of an effect on cognition, aligning with the REBUS model.

Interestingly, the decreased weight of structural priors frees up the ability for the brain to create a greater array of FC patterns and networks, supposedly enabling the bizarre and ineffable experiences associated with psychedelics (Luppi et al., 2021) [see Table 1 and Preller and Vollenweider, (2016) for the phenomenology of psychedelics]. Specifically, a reduction in FC of the mPFC (a key DMN hub) has been observed following the administration of LSD, and this area is known to be implicated in reality monitoring (Taylor et al., 2007; Subramaniam et al., 2020). Thus, a disruption in this brain region’s function may underpin and support the attenuated top-down processing, impairing an individual to accurately differentiate endogenously or exogenously generated percepts. Further evidence for the REBUS model is seen by the findings that entropy and mystical experiences involving ego dissolution (MacLean et al., 2011) have been correlated with increases in trait openness (Lebedev et al., 2016), which itself is associated with creativity, intelligence, and even increased grey matter in the inferior parietal lobule (Schretlen et al., 2010; Taki et al., 2013). Long-term meditators have decreased DMN FC (Brewer et al., 2011; Brewer and Garrison, 2014; Millière et al., 2018), with psychedelic and meditative states sharing some key characteristics such as heightened levels of perceptual sensitivity, ego dissolution, and decreased negative rumination (Brewer et al., 2011; Millière et al., 2018). Increased awareness of thoughts and feelings also seems to be a key characteristic of the ayahuasca experience and may explain the long-term thinning of the PCC in experienced users (Bouso et al., 2015). Furthermore, thinning of the PCC and thickening of the ACC, supporting DMN-TPN orthogonality, has been associated with greater levels of attention, emotional regulation, and feelings of self-transcendence, which is positively correlated with well-being and negatively associated with depression and end-of-life anxiety (Reed, 2008; Bouso et al., 2015).

#### Cortico-Striatal-Thalamo-Cortical Model (CSTC)

The CSTC postulates that 5-HT_2A_ receptor activation leads to alterations in the CSTC circuitry, resulting in disinhibition of the thalamus and reduced sensory gating, thereby increasing the amount of sensory information reaching the cortex (Vollenweider et al., 1997). Evidence for this model comes from Preller et al. (2019), who found that LSD increased excitatory connections from the thalamus to the PCC and reduced effective connectivity from the ventral striatum to the thalamus. These connectivity patterns suggest that LSD increases bottom-up informational flow by reducing thalamic-sensory gating, lending credence to the CSTC model.

#### Cortico-Claustro-Cortical (CCC) Model

The third model is known as the cortico-claustro-cortical model. The claustrum is situated between the insula and putamen and is a thin, curved sheet of neurons embedded in white mater. It contains a large density of 5-HT_2A_ receptors and bidirectional glutamatergic connections to most of the cortex. The CCC model proposes psychedelic effects are a function of activating receptors in the claustrum [the claustrum also contains k-opioid receptors, the primary target of Salvia A (Stiefel et al., 2014)], which leads to a disruption in higher cortical networks through CCC circuits that may underpin the neural and subjective effects that have been associated with the psychedelic experience. Evidence for this model is supported by the findings from Barrett et al. (2020b) (Table 2). Barret and colleagues observed that psilocybin reduced the BOLD signal of the claustrum, which was also predictive of the participant’s subjective experience.

### Limitations of REBUS, CTSC, and CCC Models

The REBUS model needs to be clearer as to what constitutes higher and lower brain regions and would benefit from dividing key brain regions (for example the hippocampus) into its constituent nuclei. Furthermore, functional outcomes of increased entropy (and region-specific entropy) are not entirely clear. For example, a recent study showed that individuals with Major Depressive Disorder (MDD) had increased entropy in bilateral hippocampi (Xue et al., 2019) and it does not seem that MDD patients have a ‘richer subjective experience’ and tend to have a more rigid rather than dynamic cognition (Carhart-Harris et al., 2017). Finally, the majority of studies supporting the REBUS model employ a seed-based approach, which assumes dysconnectivity across homogeneous brain structures, regions and networks. Thus, this presupposition has an a priori connectivity bias which limits the measurement of pharmacologically induced connectivity alterations in unaccounted brain regions and networks (Preller et al., 2018). However, it is promising that Preller et al., (2018) who used the data-driven Global Brain Connectivity (GBC) approach, recapitulated connectivity patterns from previous seed-based analyses.

The CSTC model is also incomplete because it does not account for the “efference copies” that also interact with sensory and predictive cortices (Pynn and DeSouza, 2013). Additionally, like the REBUS model, the CSTC model would be strengthened by a revision that includes more specificity regarding the models’ neuroanatomical constituents (e.g., difference in efferent and afferent output of various thalamic nuclei). Finally, the CCC model is the most recent and thereby has the least amount of support, and as newer technologies emerge this will facilitate research investigation into claustrum connectivity (because the claustrum is naturally hard to measure) in response to psychedelic drugs, which will enable this model’s validity and reliability to be further evaluated.

It is important to discuss that these models are not necessarily distinct from one another and that they are all likely involved in mediating psychedelic therapeutic effects. For instance, the feedforward thalamocortical loop that occurs due to increased serotonergic activity (5-HT_2A_ receptor agonism in the CSTC circuit), which leads to “sensory overload” and ego-dissolution, is compatible with the increased bottom-up information flow, relaxed priors, and entropy findings that support the REBUS model. Additionally, it is also possible that 5-HT_2A_ receptor activation within the claustrum leads to CSTC alteration and DMN modulation, increasing entropy and brain plasticity (Barrett et al., 2020b). Hence, it may be appropriate for psychedelics researchers to strive for a unifying psychedelic theory where various theoretical and empirical models are simultaneously tested and juxtaposed with one another. This will enable a more integrated mechanistic understanding of psychedelics’ mode of action; we can potentially understand the similarities and differences of these models and how they relate to the complex phenomenology and clinical outcomes associated with psychedelics.

### Current Challenges and Limitations of the DMN

This article rests on the fundamental premise that the DMN is an inherently valuable and meaningful network; however, this presupposition is not without challenge (Fair et al., 2008). It has been argued that the DMN is plagued by the methodological confounder that certain brain regions show intrinsic structural connectivity through vascular coupling rather than functional connectivity (Andrews-Hanna, 2012). However, the assessment of the DMN is not limited to measurements of BOLD fMRI; it has also been assessed through positron emission tomography, which measures glucose metabolism via radioactive tracers (Raichle, 2015). In addition, the neuronal activity of the DMN can be estimated by the electrical activity and magnetic fields associated with this electrophysiological activity via EEG (Foster and Parvizi, 2012) and MEG (Brookes et al., 2011), respectively.

Additionally, Morcom and Fletcher (2007) question the assumption that the DMN reveals substantial information about cognition; however, these early criticisms were based on the simple subtraction design utilized by Raichle et al. (2001) and the scarcity of literature at the time. Furthermore, Morcom and Fletcher concede that controlled experimental manipulations are necessary to further explore the DMN, and the unique experimental conditions that meditation and psychedelics offer have addressed exactly this. Over a decade later, there is a surge in publications suggesting the DMN can offer insight into the variability of human cognition (Brewer et al., 2011; Palhano-Fontes et al., 2015; Raichle, 2015; Mohan et al., 2016; Speth et al., 2016; Smigielski et al., 2019; Zhang and Volkow, 2019; Marstrand-Joergensen et al., 2021; Smallwood et al., 2021; Yeshurun et al., 2021). Furthermore, the DMN has continuously been correlated with the sense of self, ego dissolution, top-down cognitive processes (executive function), cognitive flexibility, awareness, and a number of neuropsychiatric conditions (Andrews-Hanna et al., 2014; Lebedev et al., 2015; Palhano-Fontes et al., 2015; Mohan et al., 2016; Carhart-Harris et al., 2018; Smigielski et al., 2019). For instance, a review by Barrett and Griffiths (2017) hypothesized that decreased activity and FC within DMN hubs such as the PCC and mPFC on administration of classical psychedelics are key facets that mediate the mystical experience via decreased self-referencing and disintegration in the feeling of self. These authors further posit that decreased activity and FC in the inferior parietal lobule (a lateral node of the DMN) is responsible for the feeling of timelessness and spacelessness that often governs the psychedelic experience. Therefore, although Morcom and Fletcher provided valid critiques at the time (2007), current evidence suggests the DMN is a revealing and insightful network that should remain in the purview of cognitive neuroscience and psychedelic research.

Psychedelics are not the only class of drug to alter the DMN. Alcohol also reduces FC within the DMN (Fang et al., 2021). Indeed, this decrease in resting state FC can explain 33% of the variance in alcohol craving in individuals with alcohol use disorder, thereby showing that DMN connectivity profiles/signatures may act as a possible biomarker for this condition (Fede et al., 2019). Fang et al. (2021) found that moderate alcohol consumption acutely and significantly decreased FC within the right hippocampus and right medial temporal gyrus. Unlike psychedelics, alcohol did not significantly affect the inter-network connectivity of the DMN with other cortical networks. Therefore, the acute disruption in intra-network connectivity of the DMN induced by both alcohol and psychedelics may in part explain the euphoric experience characteristic of intoxication with both drugs. The ability of psychedelics to increase global integration and connectivity (via increased internetwork connectivity) juxtaposed with its neuroplastic properties may explain its unique capacity to produce positive therapeutic health outcomes. Interestingly, it does seem that ketamine has the capacity to decrease FC within the DMN (at doses that alter consciousness) and alter the FC between the DMN and other networks (Bonhomme et al., 2016), and it has been argued that neuroplasticity is a convergent mode of action between psychedelics and ketamine (via mechanisms described above) (Aleksandrova and Phillips, 2021). Additionally, findings from Doss et al. (2020) showed that the k-opioid receptor agonist and atypical dissociative Salvinorin A decreased within network FC and increased between network FC. Static and entropic functional connectivity were best predicted by the DMN, questioning the specificity of the entropic brain hypothesis to the classical psychedelics. It is unclear if Salvinorin A elicits the same neuroplastic properties as ketamine and the classical psychedelics and whether this relates to their intra- and internetwork modulation capacity.

Table 2 outlines and summarizes the limitations of each study included in the systematic review, so a brief overview of the common limitations will be discussed. The majority of included studies were, for ethical reasons, conducted on people with prior psychedelic experience and were of small sample sizes. Therefore, it is encouraged that future researchers report effect sizes, as a power analysis by McCulloch et al., (2022) found that sample sizes greater than 60 are needed for adequately powered studies. Strikingly, only 2 of 28 papers had 60 or more participants (Mason et al., 2020, 2021). Furthermore, a number of studies included new analysis on two previously small sample datasets (Carhart-Harris et al., 2012, 2016), which is problematic for a number of reasons. The primary limitation from this sort of analysis is that one would expect that in the absence of any serious fundamental methodological flaw in the dataset that different analytical methods should support the same hypothesis. Thus, we emphasize that these findings are not necessarily independent evidence, though the fact that different analytical methodologies generated similar conclusions is reassuring. Future research should aim to carry out similar analysis on novel datasets, which would provide more robust and reliable evidence for the REBUS and entropic brain hypothesis.

The problem of inferring psychological processes or cognition from patterns of activation via neuroimaging technologies is known as reverse inference. It is not entirely clear as to whether alterations in DMN activity and connectivity are merely a by-product or epiphenomenon of psychedelics or if they play a mediating role in psychedelics’ specific psychological effects and therapeutic benefit. Thus, researchers should be tentative in drawing causal conclusions from correlative evidence.

With any field of research there is a risk of publication bias. In the context of the papers reviewed in this article, when initial articles linking the DMN to various cognitive outcomes were established in prestigious journals (refer Figure 3, Carhart-Harris et al., 2012, 2016, and Palhano-Fontes et al., 2015), it directs the field of psychedelic research and expected findings and hypotheses generated by researchers. Thus, we urge the scientific community to remain wary of overly enthusiastic claims and consider theories as parsimonious models rather than established facts. Pre-print journals such as bioRxiv and PsyArXiv can also be useful in sharing studies that may have difficulty getting published elsewhere due to negative results but can still contribute to the corpus of scientific knowledge. However, it is important to consider that due to the renewed interest in psychedelic medicine, publications with negative findings are still likely to be published and the currently published data have all been attached to registered trials.

### Potential Future of DMN Modulation–Focused Psychedelic Therapies

Studies have highlighted the potential application of the intrinsic FC of the DMN as a biomarker. The DMN has been utilized as a biomarker for attention deficit hyperactivity disorder, early antidepressant response, chemotherapy-related brain injury, depression, epilepsy, Parkinson’s disease (Yanbing et al., 2020), bipolar affective disorder, and schizophrenia (Meda et al., 2014). Using resting and task-influenced DMN activity as a cognitive biomarker is aligned with the research domain criteria of the National Institute of Mental Health. Research domain criteria is a research framework for investigating mental disorders and places an emphasis on using different biological and cognitive markers as transdiagnostic tools. Therefore, using the DMN as a biomarker of neuropsychiatric conditions can help overcome many of the limitations often associated with diagnosing these pathologies via symptomatology. These include heterogeneity of symptoms for the same condition and less reliance on the subjective judgment of a clinician.

The REBUS and predictive-coding models may offer explanatory power in terms of psychedelics transdiagnostic potential. Neuropsychiatric conditions are characterized by distinct DMN signature abnormalities, which psychedelics may therapeutically normalize. Little is known regarding how psychedelics influence top-down and bottom-up information-processing streams and whether these pathways converge upon the DMN. Future research might take Bayesian approaches to determine if psychedelics alter hierarchical sensory processing and if this may contribute to updating models of the self and world as proposed in the REBUS model. Moreover, belief system updating may alter top-down processing, with greater cognitive flexibility modulating distinctions of exogenous and interoceptive information. These hypotheses can be directly assessed in future research by examining how nodes of the DMN may modulate thalamic activity via effective connectivity analyses, and vice versa, to gain a deeper understanding about information transfer as demonstrated by Preller et al. (2019).

Micro-dosing of psychedelics (particularly LSD and psilocybin), which generally involves taking sub-hallucinogenic doses, has been reported to lead to decreased mind-wandering (Polito and Stevenson, 2019), which at the neural level is reflected by reduced DMN activity. For instance, a double-blinded placebo-controlled study by Murray et al., (2022) administered low doses of LSD (13 or 26 μg sublingual). Using EEG, scalp electrodes were placed on the midline of the brain to infer source localization for key DMN regions and showed a reduction in broadband oscillatory power, consistent with findings using larger doses. Across all EEG frequencies (delta, theta, alpha, beta, and gamma), the 26-μg dose saw greater reductions in oscillatory power compared with the 13-μg dose. There is, however, currently no direct evidence or clinical trials that show how micro-dosing modulates the DMN, and this should be explored in subsequent research. This can illuminate how much DMN modulation (and its potential downstream benefits) is dependent on the rich subjective experience associated with larger doses of psychedelics. Furthermore, studies could aim at determining a “minimum effective dose,” which would likely cause fewer challenging experiences that may result from administration of high-dose psychedelics. The same rationale also applies to non-hallucinogenic psychedelic analogues, which are currently being explored and have shown anti-depressant effects, at least in rodent models (Cao et al., 2022). However, it is important to note that challenging experiences may also be conducive of positive therapeutic outcomes (Yaden and Griffiths, 2020).

A worthwhile future direction would be the comparison of DMN modulation for experienced psychedelic users and psychedelic naïve individuals because it would help decipher the interaction between the acute and enduring effects of these substances on this network. Alternatively, well-designed longitudinal research involving several scanning sessions could clarify how modulations of DMN FC may reflect the dynamics of lasting effects of psychedelics in brain activity as demonstrated by McCulloch et al. (2022). Interestingly, in this recent study within and between DMN FC differences were not significantly (with low effect size, d = 0.2) modulated at 1-week and 3-months post injection. This study design could be replicated with a greater sample size and additional acute time points to further delineate the acute and enduring connectivity patterns of psychedelic drugs.

Clinically, psychedelics could be used in conjunction with psychotherapeutic techniques such as mindfulness meditation. Mindfulness and psilocybin retreats have shown promising outcomes for people with depression (Carhart-Harris et al., 2017). It seems that mindfulness and psilocybin therapy modulate the DMN in similar ways (a reduction in FC and activity within the DMN) and may act synergistically. This may be because mindfulness, which acts to increase and cultivate non-judgmental awareness of one’s thoughts and feelings, may help facilitate a positive psychedelic experience and vice versa.

Additionally, a legitimate research question in the context of depression is whether infrequent psilocybin treatment (i.e., quarterly) juxtaposed with standard frontline treatments such as cognitive behavioral therapy and selective serotonin reuptake inhibitors such as escitalopram may yield a superior continued reduction in depressive symptomatology. This type of study design could be within the purview of future research scientists and, if effective, could be translated to a variety of other neuropsychiatric conditions. Furthermore, it is important that clinical (and pre-clinical) research promotes a “bench-to-bedside” approach; with scheduled drugs such as psychedelics, there will be a need for government lobbying and policy reform [see Marks and Cohen, (2021) for a roadmap for psychedelic therapy].

## CONCLUSIONS

This systematic review provides evidence to support the notion that classical psychedelics are capable of modulating the DMN, which is correlated with ego dissolution, increased brain entropy, and improved mental health and well-being. Our review of the data shows psychedelics are a valuable tool for investigating this network and considers the potential therapeutic implications of this effect. Psychedelics are showing promise as tools to aid our understanding of the brain and mind in greater detail. Finally, this understanding can be harnessed to treat a variety of neuropsychiatric conditions and potentially increase healthy individuals’ psychological well-being.

## Interest Statement

J.S. and D.P. are directors of a not-for-profit medicinal psychedelics research institute, which receives funding from commercial sources to facilitate research in this field. D.C. has received grant monies for research from Servier, Boehringer Ingelheim; Travel support and honoraria for talks and consultancy from Servier, Seqirus, Lundbeck. He is a founder of the Optimal Health Program (OHP) and holds 50% of the IP for OHP and is part owner of Clarity Healthcare. He does not knowingly have stocks or shares in any pharmaceutical company. D.C. is an Advisory Board Chair of a not-for-profit institute specializing in psychedelic medicines research. D.N. is a consultant to COMPASSPathways, Neural Therapeutics, Beckley PsyTec, and Awaknlifesciences. S.R. is employed at a not-for-profit medicinal psychedelics research institute, which receives funding from commercial sources to facilitate research in this field. All other authors have no conflicts of interest.
